# Development and Characterization of Whey Protein-Based Nano-Delivery Systems: A Review

**DOI:** 10.3390/molecules24183254

**Published:** 2019-09-06

**Authors:** Ho-Kyung Ha, Scott A. Rankin, Mee-Ryung Lee, Won-Jae Lee

**Affiliations:** 1 Department of Animal Science and Technology, Sunchon National University, Suncheon 57922, Korea; 2 Department of Food Science, University of Wisconsin, Madison, WI 53706, USA; 3 Department of Food and Nutrition, Daegu University, Gyeongsan 38453, Korea; 4 Department of Animal Bioscience (Institute of Agriculture and Life Science), Gyeongsang National University, Jinju 52828, Korea

**Keywords:** *β*-lactoglobulin, nano-delivery system, nanoemulsion, nanoparticle, whey protein

## Abstract

Various bioactive compounds (BCs) often possess poor stability and bioavailability, which makes it difficult for them to exert their potential health benefits. These limitations can be countered by the use of nano-delivery systems (NDSs), such as nanoparticles and nanoemulsions. NDSs can protect BCs against harsh environments during food processing and digestion, and thereby, could enhance the bioavailability of BCs. Although various NDSs have been successfully produced with both synthetic and natural materials, it is necessary to fulfill safety criteria in the delivery materials for food applications. Food-grade materials for the production of NDSs, such as milk proteins and carbohydrates, have received much attention due to their low toxicity, biodegradability, and biocompatibility. Among these, whey proteins—from whey, a byproduct of cheese manufacturing—have been considered as excellent delivery material because of their high nutritional value and various functional properties, such as binding capability to various compounds, gelation, emulsifying properties, and barrier effects. Since the functional and physicochemical properties of whey protein-based NDSs, including size and surface charge, can be key factors affecting the applications of NDSs in food, the objectives of this review are to discuss how manufacturing variables can modulate the functional and physicochemical properties of NDSs and bioavailability of encapsulated BCs to produce efficient NDSs for various BCs.

## 1. Introduction

Nano-delivery systems (NDSs) can be defined as vehicles of submicron size, ranging from 1 to 200 nm, which can encapsulate and protect bioactive compounds (BCs) and nutrients [1,2]. The use of NDSs has become a promising tool to increase the bioavailability of entrapped BCs because the small size of NDSs, together with their large surface area, may offer prolonged gastrointestinal residence time and improve the mucosal adhesion to small intestine and the interaction with gut cells [2,3,4]. Although both synthetic and natural materials have been successfully used to develop NDSs, the use of synthetic materials may not be appropriate for the application to foods due to the potential toxicity problem and demand for generally recognized as safe (GRAS) ingredients [5,6]. Therefore, it is highly recommended that food-grade materials are used for the production of NDSs. Among various food-grade materials, whey proteins have great potential as delivery material, since whey proteins are known to sustain high nutritional values and various functionalities [6,7].

Whey proteins are derived from whey, a byproduct of cheese production, and have received increasing attention as ideal materials for NDSs because of their GRAS status, low cost, great nutritional value, and various functionalities [8,9]. Whey proteins are composed of various globular proteins, including *α*-lactalbumin (*α*-la), *β*-lactoglobulin (*β*-lg), bovine serum albumin (BSA) and immunoglobulins, and lactoferrin [10,11]. In particular, *β*-lg comprises about 50%–60% of whole whey proteins in cow milk and contains one free thiol residue and two disulfide bonds [7,8]. The most widely produced whey protein products are whey protein concentrates (WPCs) and whey protein isolates (WPIs). WPC is obtained by the ultrafiltration of whey and contains about 50%–75% total proteins, whereas WPI is composed of higher protein concentrations exceeding 90%. Further processing, including diafiltration or ion exchange, is necessary to produce WPI [7,12].

The functional properties of whey proteins and manufacturing methods of whey protein-based NDSs have been reported in previous studies [5,13]. Many review papers focused on the physicochemical properties of NDSs [5,7,13]. However, the modulation of physicochemical properties of whey protein-based NDSs, especially using mild heat-induced gelation and chemical modification processes, and their impacts on the delivery of BCs and food applications, have not been extensively studied. This review is focused on the major advantages of whey protein as a delivery material and describes how manufacturing processes could modulate the physicochemical properties of whey protein-based NDSs. In addition, the major advantages of whey protein-based NDSs for food applications are reviewed.

## 2. Functional Properties of Whey Protein as a Delivery Material

**Figure 1:**
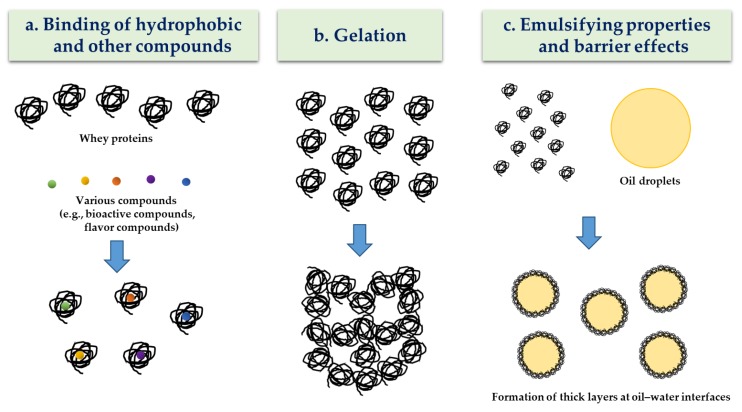
Schematic representation of functional properties of whey proteins as a delivery material. Adapted and modified from Livney et al. [7].

Whey protein as a delivery material has several functional properties, such as the ability to bind to hydrophobic BCs and other compounds, gel-forming capacities, emulsifying properties, and barrier effects (Figure 1). An understanding of their functional properties can be very useful for the manufacture of whey protein-based NDSs, which have enhancing capabilities for the protection and delivery of BCs during food processing, storage, and digestion. Moreover, the use of whey protein, such as *β*-lg, is beneficial for the oral delivery of BCs since the pepsin resistance of native *β*-lg in the stomach can be utilized for the effective protection of entrapped BCs against the harsh gastric environment [10,14].

### 2.1. Binding Ability to Hydrophobic BCs and Other Compounds

One of the most important factors for the ideal delivery material is the binding ability to target BCs. *β*-lg is known as a member of the lipocalin protein family and has three potential binding sites for hydrophobic compounds: the inner hollow of the *β*-barrel, the surface cavity in a channel between the *α*-helix and *β*-barrel, and the external surface near Trp19–Arg124 [11,15,16]. Numerous studies suggest that whey protein, mainly *β*-lg, could bind various hydrophobic BCs [7], such as vitamin D [1,17], retinol [18,19], polyphenols [16,20], and fatty acids [21,22], which make it an ideal delivery material for NDSs. Since hydrophobic attraction is the main mode of interaction between *β*-lg and hydrophobic BCs [11], increasing the hydrophobicity of *β*-lg can lead to an increase in its binding affinity and encapsulation efficiency for hydrophobic BCs [22,23,24]. Structural changes, including the heat-induced partial unfolding of *β*-lg, may lead to exposure of the hydrophobic residues buried inside *β*-lg, which can result in an increase in the surface hydrophobicity of *β*-lg [23,25]. It was reported that an increase in the surface hydrophobicity of whey proteins resulted in an increase in the encapsulation efficiency of hydrophobic BCs, such as coenzyme Q10 [23], quercetin [24], and the omega-3 fatty acid docosahexaenoic acid (DHA) [22], in whey protein-based NDSs.

Electrostatic interactions between whey proteins and hydrophobic BCs also affect binding affinity [17]. One of the manufacturing variables, pH, may affect these electrostatic interactions and could be an important factor in mediating the ability to bind hydrophobic BCs. The effects of pH on the binding of hydrophobic BCs were studied by Forrest et al. [17] using vitamin D_3_. The EF loop region of *β*-lg, a helix–loop–helix structural domain spanning residues 85–90, acts as a gate for the internal binding site for hydrophobic BCs, and changes from a closed to an open position conformation in the pH range of ~6.2–8.2 [17,26]. It was observed that 1 mol *β*-lg could bind approximately 1 mol vitamin D_3_ at pH 2.5, where the EF loop exhibits a closed conformation with internal binding sites unavailable. However, the binding of *β*-lg with vitamin D_3_ was increased to 1.36 mol vitamin D_3_/mol protein at pH 6.6 and 1.54 mol vitamin D_3_/mol protein at pH 8.0, since the EF loop shifted to an open position allowing vitamin D_3_ to access the internal binding site of *β*-lg at this pH range [17]. The binding affinity of retinol to *β*-lg was also slightly diminished as pH was decreased from 7.0 to 5.2 [19]. The binding ability of whey proteins with hydrophobic BCs, including vitamin D_3_ and retinol, could make them effective and reliable delivery materials for encapsulating and protecting hydrophobic BCs [5,27].

Due to their ability to bind hydrophobic flavor compounds, whey proteins have also been used as a delivery material for enhancing flavor or masking off-flavor [28,29,30,31]. Thermodynamic studies on the binding of flavor compounds to several food proteins showed that the Gibbs free energy of whey proteins was lower than that of casein and soy proteins. This indicates that whey protein had higher binding affinity for flavor compounds, such as vanillin, than soy protein and casein [32]. *β*-lg can also bind various flavor compounds, such as alkanes, aldehydes, ionones, lactones, and esters [33,34,35]. BSA was reported to bind alkanes [36,37,38,39], aldehydes, and ketones [40,41], and *α*-la to bind aldehydes and methyl ketones [42].

### 2.2. Gelation

Whey proteins retain excellent gel-forming capability, and have been used as gelling agents in the food industry [43]. The heat-induced gelation of whey proteins is a traditional gelation method for the preparation of whey protein-based NDSs. It can be generally achieved by the heating of whey protein solution or food containing whey above 80 °C [44]. In addition, a heat treatment with high temperatures above 100 °C is used in the spray-drying process, which can be the most common technique for the production of delivery systems [45]. During heat treatment, whey proteins can be partially or fully unfolded, contributing to the exposure of hydrophobic groups and free sulfhydryl groups. This may enhance hydrophobic interactions and the formation of disulfide bonds between whey protein molecules, resulting in the formation of gel networks, including NDSs of various sizes [10]. However, there is a limit regarding the use of conventional heat-induced gelation method and spray-drying for forming whey protein-based NDSs since heat treatment may result in the loss of heat-sensitive BCs and nutrients [46]. This problem can be solved by the use of cold-set gelation [23,47], a sub-ambient temperature process [22,24], or a two-step temperature process [25]. 

The enzyme-induced cross-linking of proteins is an easy and simple method for producing NDSs without using heat treatment, and has less toxicity concerns than that of chemical cross-linking (e.g., glutaraldehyde) [48]. Food-grade transglutaminase catalyzes acyl transfer reactions between the γ-carboxyl groups of glutamine moieties and various primary amines, including the ε-amino groups of lysine moieties of proteins, which can induce the cross-linking of proteins [48]. Since native *β*-lg is not sensitive to transglutaminase-induced cross-linking, possibly due to the buried state of glutamine and lysine moieties inside [49], the heat-induced partial unfolding of *β*-lg can be necessary to induce the aggregation of whey proteins by transglutaminase [50]. The use of transglutaminase on WPI pre-heated at 80 °C for 15 min promoted the aggregation of whey proteins, resulting in the formation of nanoscale particles ~37 nm in size. Transglutaminase-induced cross-linking of *β*-lg was strongly affected by enzyme concentrations [51]. Sodium dodecyl sulfate polyacrylamide gel electrophoresis (SDS-PAGE) analysis of *β*-lg showed that increasing the concentration of transglutaminase from 10 to 25 unit/g led to a decrease in the monomeric and dimeric forms of *β*-lg, indicating that more extensive cross-linking in between *β*-lg molecules was formed at higher concentrations of enzyme [51].

### 2.3. Emulsifying Properties and Barrier Effects

Whey proteins have been considered to be good emulsifiers because whey proteins can adsorb at oil–water interfaces and produce thick layers, contributing to the stabilization of emulsion droplets and preventing lipid separation which works against coalescence [52]. Native *β*-lg is a rigid and inflexible globular protein with lower surface activity than other flexible milk proteins, such as caseins [23]. However, the emulsifying properties of *β*-lg can be improved by heating above 60 °C before emulsification, which can induce the partial unfolding of *β*-lg, leading to the surface exposure of reactive groups, such as hydrophobic residues and free sulfhydryl groups. This may contribute to additional adsorptions of whey proteins to the oil–water interfaces, which can thus stabilize emulsions [23,53]. Pre-heated whey protein has been used as an emulsifier for both single (e.g., oil-in-water, O/W) [23,50] and multiple emulsions (e.g., water-in-oil-in-water, W/O/W) [53,54,55].

Since whey proteins have antioxidant activity, metal chelating ability, and gel-forming capability, whey proteins used as delivery material may provide barrier effects for enhancing the encapsulation efficiency of BCs and preventing BC oxidation. It has been reported that *β*-lg acts as a free radical scavenger, since it has free sulfhydryl residues and aromatic amino acids leading to antioxidant activity [56,57]. It can thus be used to enhance the oxidative stability of BCs [58,59]. In addition to the antioxidant activity and metal chelating property of whey proteins, whey proteins used as materials in nanoemulsion delivery systems can act as barriers on fat–water interfacial areas [60,61]. They can contribute to a decrease in the access of transition metals to lipid droplets, resulting in a reduction in lipid oxidation [55]. It was reported that the encapsulation of fish oil in whey protein-based NDSs, such as WPC multiple nanoemulsion, led to a decrease in the formation of primary and secondary oxidation products and the development of fishy off-flavor compounds [55]. Moreover, whey protein-based ND, which was based on *β*-lactoglobulin/oleic acid-modified chitosan oligosaccharide nanoparticles, enhanced the oxidative stability of docosahexaenoic acid (DHA) during storage in skim milk [22,55].

## 3. Physicochemical Properties of Whey Protein-Based NDSs

**Figure 2:**
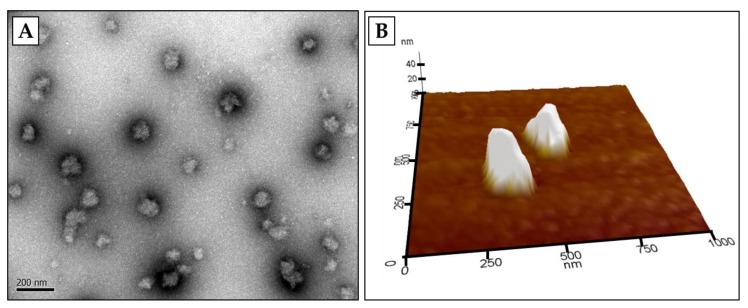
Microscopic images of *β*-lg nanoparticles observed in transmission electron microscopy (TEM) (**A**) and atomic force microscopy (AFM) (**B**). *β*-lg nanoparticles were prepared using the modified ionic gelation method described in Ha et al. [24]. This figure is original and has not been previously published. Scale bar = 200 nm.

Physicochemical properties, such as particle size, size distribution, and surface charge, are important factors that impact the functional properties of NDSs [55,56,57,62,63,64]. Various electron microscopy techniques, such as transmission electron microscopy (TEM), scanning electron microscopy (SEM), and atomic force microscopy (AFM), have been used to determine the morphology, size, and aggregation state of NDSs. Examples of the microscopic images of whey protein-based NDSs are presented in Figure 2, where spherically shaped *β*-lg nanoparticles with a size ranging from ~50 to 200 nm were observed.

Dynamic light scattering (DLS) is the most commonly-used technique for measuring the hydrodynamic radius and size distribution profile of NDSs. Scattered light is used to determine the rate of particle movement caused by Brownian motion. A hydrodynamic radius is calculated using the Stokes–Einstein equation, while the size distribution of NDSs can be expressed as the polydispersity index, which ranges from 0 (monodispersed) to 1 (polydispersed) [65]. On the other hand, the surface charge of NDSs can be evaluated through zeta-potential value. The zeta-potential value of NDSs can be measured with laser Doppler velocimetry (LDV). In the LDV measurement, the velocity of a moving NDS in an applied electric field is determined using a laser, and is proportional to electrophoretic mobility. The electrophoretic mobility value can be used to calculate the zeta-potential value using the Smoluchowski equation [66].

**Figure 3:**
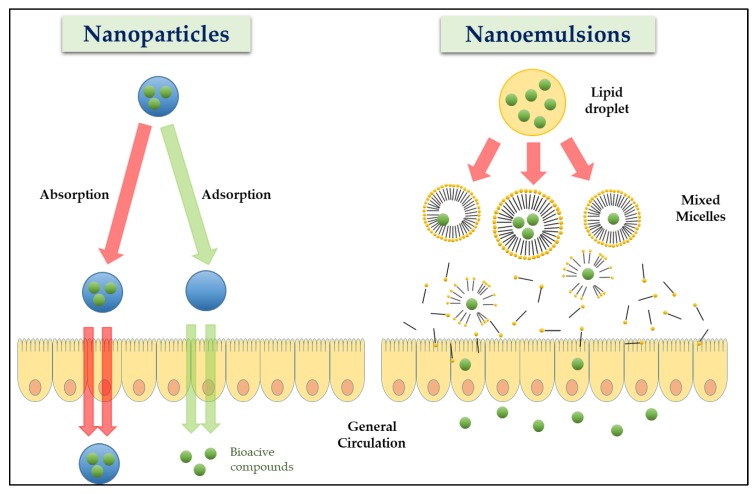
Schematic representation of different absorption mechanisms of bioactive compounds encapsulated in nanoparticles and nanoemulsions. Adapted and modified from Chen et al. [6].

It has been known that the physicochemical properties of NDSs are important factors affecting the nutritional efficacy of BCs entrapped in NDSs. The term bioavailability is the crucial phrase of nutritional efficacy, and is defined as “the rate and extent to which the active substances or therapeutic moieties contained in a drug are absorbed and become available at the site of action” [67]. On the other hand, the terms uptake and bioaccessibility have also been used for BCs and nutrients (Figure 3) [6]. The term uptake (or intestinal absorption) is defined as “the fraction of the dose that is absorbed through the intestinal walls”[3], while the term bioaccessibility refers to “the fraction of an ingested nutrient that is released from food matrix and is available for absorption in the gut after digestion (typically based on in vitro procedures)” [67].

### 3.1. Impacts of the Physicochemical Properties on the Bioavailability of BCs and Physical Stability of NDs 

#### 3.1.1. Size

The particle size of NDSs can be a crucial factor affecting their cellular uptake. It is believed that a reduction in particle size may increase the cellular uptake of NDSs, since the surface area of the NDSs which can interact with intestinal cells would increases with a decrease in the particle size [62,68,69]. Ha et al. [4] investigated the direct uptake of *β*-lg nanoparticles in human intestinal epithelial (Caco-2) cells. Caco-2 cells have been commonly used as an intestinal cellular model for human intestinal epithelium, and have been used to investigate the absorption of BCs, drugs, and delivery systems. It was reported that the size of *β*-lg nanoparticles was negatively correlated (*r* = −0.76) with their cellular uptake in Caco-2 cells, indicating that those whey protein-based NDSs with smaller particle sizes could be very useful delivery systems for improving the cellular uptake of BCs in comparison with delivery systems with larger particle sizes [4]. The subcellular size of NDs (e.g., less than ~200 nm) contributes to the larger surface area available for interaction with the mucosa of the small intestine, which can lead to an increase in the cellular uptake of NDSs [3].

**Figure 4:**
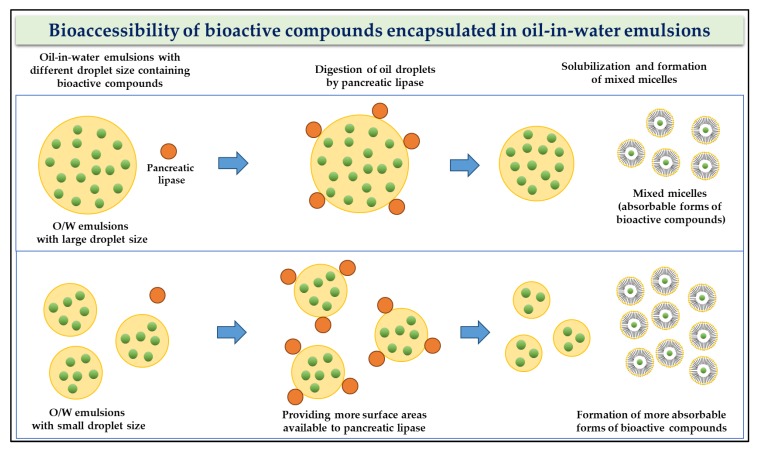
Illustration of the impacts of droplet size on the digestion of oil droplets and bioaccessibility of bioactive compounds encapsulated in oil-in-water emulsions. Adapted and modified from Zou et al. [73].

When nanoemulsion was used as a delivery system, a reduction in the droplet size of the nanoemulsion could enhance the bioaccessibility of encapsulated BCs during gastrointestinal digestion (Figure 4) [70,71,72,73]. During the digestion of oil-in water (O/W) emulsions in the gastrointestinal tract, the oil droplets of emulsions containing hydrophobic BCs can be hydrolyzed into free fatty acids because of the presence of pancreatic lipase and the release of hydrophobic BCs. Prior to the absorption of BCs in the small intestine, released BCs should exist in a mixed micellar form that is absorbable. Free fatty acids interact with bile salts, leading to the formation of mixed micelles in the small intestine. These mixed micelles can solubilize hydrophobic BCs and be absorbed by intestinal epithelial cells. Therefore, the fraction of mixed micelle forms of BCs after in vitro digestion, or bioaccessibility, can be used as an indicator of oral bioavailability of BCs in emulsions [70,71,72]. A reduction in the droplet size of emulsions provides more surface area available to pancreatic lipase, which can result in an increase in lipid digestion and the formation of mixed micelles with hydrophobic BCs. In our previous study, a decrease in the initial droplet size of *β*-lg nanoemulsions from 360 to 94 nm led to an increase in the bioaccessibility of the hydrophobic BC coenzyme Q10, from 39% to 57% [72], indicating that droplet size was a crucial determinant affecting the bioaccessibility of hydrophobic BC.

The conventional water-in-oil-in-water (W_1_/O/W_2_) multiple emulsion (e.g., >1000 nm) has been known as an effective delivery system since it can deliver both hydrophilic BCs in the inner water phase (W_1_) and hydrophobic BCs in the inner oil phase (O) [74]. However, the inherent thermodynamic instability of conventional multiple emulsions makes them prone to coalescence and sedimentation. Therefore, the application of these multiple emulsions to food was limited [75]. The use of a nanoemulsion as a delivery system for BCs is more advantageous over conventional emulsions since nanoemulsion delivery systems with small droplet sizes (e.g., less than ~200 nm) have increased stability against coalescence and sedimentation during food storage [23,76]. Recently, W_1_/O/W_2_ multiple nanoemulsions with sizes ranging from 190 to 210 nm were successfully produced with WPC [55] and WPC–pectin [77]. During storage at 37 °C for 15 days, the droplet size and polydispersity index of WPC multiple nanoemulsions were not significantly changed, indicating that those multiple nanoemulsions had excellent stability [55].

#### 3.1.2. Surface Charge

Since the surface charge of NDSs affects the stability of NDSs, zeta-potential value can be used as an indicator for the stability of NDSs. Higher zeta-potential values, either positive or negative, indicate higher repulsive forces between NDSs, which makes them more stable [78]. It is generally accepted that zeta-potential values above ±30 mV imply good stability, while zeta-potential values between −5 and +5 mV indicate poor stability [78]. The surface charge of NDSs can be an important factor in determining the cellular uptake of NDSs since the surface charge is strongly related to the affinity of NDSs with cell membranes [79,80,81]. The adsorption of neutral and negatively charged NDSs onto the negatively charged surface of cell membranes were much less than that of positively charged NDSs [80]. It was reported that positively charged nanoparticles can bind negatively charged groups on the cell surface through electrostatic attraction [68]. On the other hand, a reduction in the negative surface charge of NDSs can decrease the electrostatic repulsions with negatively charged cell membranes, which may enhance the affinity of NDSs to cell membranes. When the zeta-potential values of chitosan-grafted methyl methacrylate nanoparticles was increased from −40 to −15 mV, the cellular uptake of those nanoparticles in murine macrophage cells was increased [68]. A similar trend for whey protein-based NDSs was observed by Ha et al. [4], who found that there was a positive correlation with *r* values of 0.75 between zeta-potential value and cellular uptake of *β*-lg nanoparticles.

### 3.2. Modulation of Size and Surface Charge of Whey Protein-Based NDS

As discussed earlier, whey proteins undergo conformational changes depending on the environmental conditions, such as pH and temperature. The conformational changes of whey proteins may affect not only binding of whey proteins with other molecules, such as BCs and co-delivery materials (e.g., carbohydrates), but also associations between whey protein molecules [82]. The confirmation can therefore be a crucial factor determining the physicochemical properties of NDSs, including size and surface charge.

#### 3.2.1. Conventional Thermal Gelation

It is known that the heat treatment of *β*-lg using high temperatures (e.g., >80 °C) results in conformational changes of *β*-lg, including its secondary, tertiary, and quaternary structure [44,83,84]. These changes include the alteration of secondary structure and unfolding of *β*-lg that induce the exposure of hydrophobic residues and free sulfhydryl groups, buried inside, to the outer water environment. At lower whey protein concentrations, this can promote intermolecular associations between whey protein molecules via hydrophobic interactions and disulfide bond formations, leading to the production of nanoscopic gel networks [13].

WPI NDSs were manufactured using conventional thermal gelation [85]. In this study, NDSs with a size of 49.6 nm were obtained at 68.5 °C for 2 h, while heat treatment at 90 °C for 30 min resulted in the formation of NDSs with a size of 62.7 nm [85]. On the other hand, heat treatment after desolvation with ethanol was used to produce WPI NDS [86]. When heating temperature was increased from 40 to 80 °C for 5 min, the size of WPI NDSs was increased from 183 to 453 nm. Further increases in temperature, to 90 °C, resulted in the formation of larger particles with a size about 4294 nm [86]. Although the conventional thermal gelation of whey protein is a simple and easy method for manufacturing NDSs, it can induce the destruction of heat-sensitive BCs.

#### 3.2.2. Mild Heat-Induced Gelation

##### Cold-Set Gelation

Cold-set gelation can be a useful mild heat treatment process to manufacture a whey protein-based delivery system for heat-sensitive BCs, such as riboflavin [43,87] and coenzyme Q10 [23]. Cold-set gelation is usually achieved using two steps. First, the pH of whey protein solutions is adjusted to above their isoelectric point (pH~5.3) and then pre-heated, typically at 70–90 °C, for 5–60 min. Second, pre-heated whey protein solutions are cooled at room temperature and BCs are dissolved in solution. The gelation of whey protein solutions is induced by adding positive ions, such as calcium, which may increase associations between whey protein molecules [14].

In the cold-set gelation process, pre-heating temperature, pH, and salt concentrations are key factors that affect the physicochemical properties of whey protein-based NDSs. It was observed that an increase in pre-heating temperature from 60 to 70 °C resulted in a decrease in the size of *β*-lg NDSs from 220 to 169 nm, and a decrease in zeta-potential value from−6.8 to −8.8 mV [23]. Giroux et al. [44] reported that the size of whey protein NDSs decreased from >200 to ~50 nm when increasing the pH from 5.0 to 6.0. On the other hand, as calcium concentrations were increased from 3 to 5 mM, the size of *β*-lg NDSs increased from 94 to 360 nm [72].

##### Sub-Ambient Temperature Process

Sub-ambient temperature treatment below ~25 °C can be a valuable process in manufacturing whey protein-based NDSs encapsulating heat-sensitive BCs and to modulate physicochemical properties of NDSs such as size. It was reported that a reduction in the size of *β*-lg NDSs, from 319 to 186 nm, was observed when the sub-ambient temperature was decreased from 20 to 5 °C [24]. When whey protein, including *β*-lg, is heated above room temperature or cooled to below room temperature, it may partially unfold [22,24,88,89]. The partial unfolding of *β*-lg results in exposure of the hydrophobic residues and sulfhydryl groups buried inside to the watery environment. A decrease in sub-ambient temperature resulted in an increase in the surface hydrophobicity of *β*-lg due to an increase in the partial unfolding and exposure of hydrophobic residues to the surface of *β*-lg [22,24]. This could lead to an increase in the probability of intramolecular associations between *β*-lg molecules and the production of more compact and smaller NDSs by increasing ongoing fusions of *β*-lg molecules [22,24]. However, sub-ambient temperature treatment did not significantly affect the surface charge of *β*-lg NDSs. In addition, decreasing sub-ambient temperature resulted in an enhancement in the encapsulation efficiency of the hydrophobic BCs DHA and quercetin [22,24]. Moreover, the production of primary and secondary oxidation products and fishy off-flavor compounds decreased with a decrease in sub-ambient temperature [22].

##### Two-Step Temperature Process

**Figure 5:**
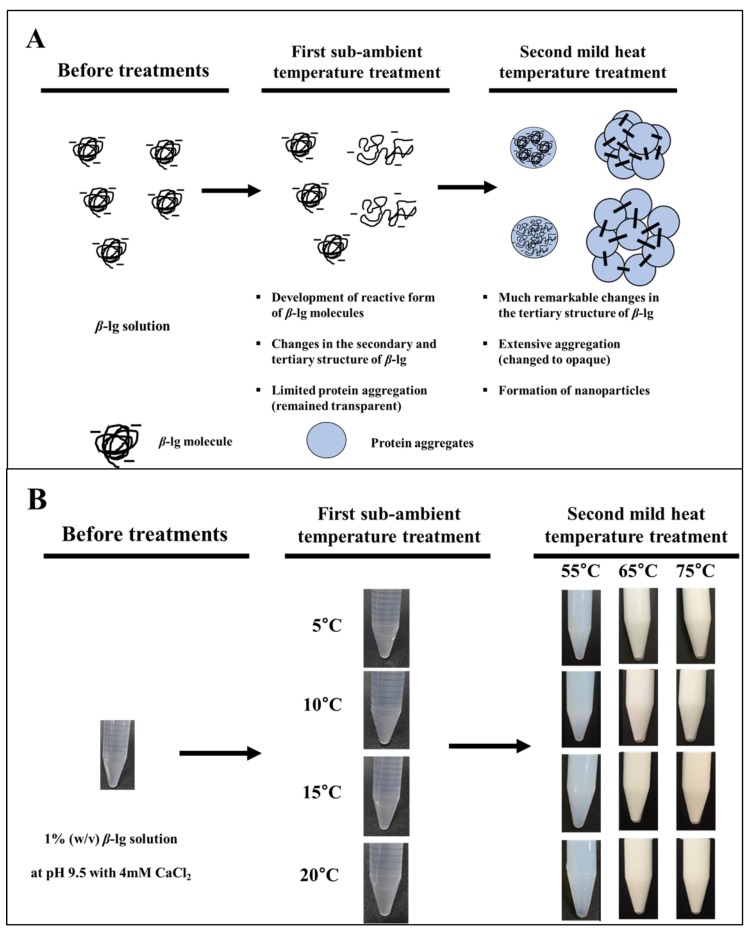
Overall schemes of the two-step temperature process used to produce *β*-lg nano-delivery systems (NDSs). (**A**) Structural changes of *β*-lg molecules during NDS formation by the use of the two-step temperature process; (**B**) changes in the turbidity of *β*-lg solution during NDSs formation by the use of the two-step temperature process. This figure was adapted, with permission, from Ha et al. [25].

In our previous study [25], *β*-lg NDSs were manufactured using a two-step temperature process at pH 9.5. This method consists of both the first sub-ambient temperature treatment from 5 to 20 °C for 30 min and the second mild heat treatment from 55 to 75 °C for 10 min to induce the structural changes of *β*-lg (Figure 5) [25]. After the second heat treatment, spherically shaped NDSs were formed, with sizes ranging from 61 to 214 nm. The size of *β*-lg NDSs was decreased with an increase in the first sub-ambient temperature from 5 to 20 °C, and a decrease in the second mild heat treatment from 75 to 55 °C. When the second heating temperature was increased from 55 to 75 °C, the zeta-potential value of *β*-lg NDSs was decreased from −13.1 to −19.3 mV. These changes in the physicochemical properties of *β*-lg NDSs can be due to the conformational changes of *β*-lg under the first sub-ambient temperature treatment and the second mild heat treatment. During the first and second temperature treatments, a notable change in the transformation of *α*-helix to *β*-sheet content and exposure of hydrophobic residues and free sulfhydryl groups was observed. Those secondary and tertiary structural changes of *β*-lg significantly affected the physicochemical properties of *β*-lg, including size and surface charge [25].

#### 3.2.3. Chemical Modification of Delivery Materials

*β*-lg and chitosan oligosaccharide (CSO) were used as delivery materials for encapsulating hydrophobic BCs, such as quercetin [24] at pH 4.4 and docosahexaenoic acid (DHA) [22] at pH 4.0. An increase in the hydrophobicity of delivery material may enhance binding affinity with hydrophobic BCs via hydrophobic attractions [89]. To enhance the binding affinity and encapsulation efficiency of those hydrophobic BCs, chemical modification with fatty acids, such as linoleic acid [24] and oleic acid [22], was used to increase the hydrophobicity of CSO. The substitution of amino groups of CSO with carboxyl groups of fatty acids by using chemical modification leads to an increase in the number of hydrophobic fatty acid residues on the surface of CSO, which may result in an increase in the hydrophobicity on the surface of CSO.

As an increase in the degree of substitution of linoleic acid-modified CSO was increased from 0% to 8.5%, the sizes of *β*-lg/CSO NDSs were increased from 258 to 351 nm [24]. Similarly, the size of *β*-lg/CSO NDSs increased with an increase in the degree of substitution of oleic acid-modified CSO. Since the substitution of fatty acids on the surface of CSO could increase hydrophobicity on the surface of CSO, intermolecular hydrophobic attractions between CSO and *β*-lg could be increased, which may lead to the formation of bigger NDSs [22].

An increase in the degree of substitution with fatty acids resulted in an increase in the encapsulation efficiency of the hydrophobic BCs quercetin and DHA, and a decrease in the autoxidation of DHA and development of fishy off-flavors [22,24]. Since an increase in the degree of substitution of CSO with fatty acid could provide more hydrophobic binding sites for hydrophobic BCs, it contributed to an increase in the binding affinity and encapsulation efficiency of hydrophobic BCs inside of *β*-lg/CSO NDSs [22,24]. Moreover, this may also lead to a decrease in the mobility within *β*-lg/CSO NDSs and the diffusion of prooxidants. Therefore, the autoxidation of DHA and development of off-flavor compounds from oxidized DHA decreased with an increase in the degree of substitution [22].

## 4. Major Advantages of Whey Protein-Based NDSs for Food Application

It is generally accepted that the encapsulation of BCs in whey protein-based NDSs can enhance the bioavailability of BCs because NDSs effectively protect BCs and increase their physical and chemical stability during food storage and digestion [1,20,21]. In addition, the application of whey protein-based NDSs to foods offers several advantages, since whey-protein based NDSs have promising prebiotic effects and can reduce off-flavor development.

### 4.1. Enhancement of Bioavailability of BCs by Encapsulation

An increase in the encapsulation efficiency of BCs in delivery systems, including NDSs, may lead to an increase in their bioavailability [90]. Since most hydrophobic BCs have poor water solubility and stability under the harsh environments faced in food processing (manufacturing and storage) and gastrointestinal digestion, they can easily lose biological activity [14]. It was reported that the use of multiple nanoemulsion delivery systems to encapsulate fish oil resulted in a decrease in the formation of primary and secondary oxidation products during storage for 15 d [55]. This indicates that the encapsulation of BCs in NDSs can be a promising tool to improve the oxidative stability and biological activity of encapsulated BCs, thereby improving the bioavailability of BCs.

When whey protein/zein NDSs with a size ranging from 200 to 250 nm were used to encapsulate drugs, this led to an increase in the oral bioavailability of lopinavir and fenretinide by four- and seven-fold, respectively, compared to those of free drugs in BALB/c mice [91]. Whey protein/zein NDSs enhanced the stability and cellular uptake of drugs in intestinal epithelial cells. In addition, since whey protein/zein NDSs were strongly mucoadhesive, encapsulation in NDSs could prolong the gastrointestinal residence time of drugs, which could contribute to an increase in the oral bioavailability of lopinavir and fenretinide [91].

### 4.2. Potential Prebiotic Effects

It was reported that whey protein-based NDSs prepared with a prebiotic, such as inulin, exhibited potential prebiotic effects. WPI and inulin were used to produce NDSs for the encapsulation of the hydrophobic BC resveratrol. An increase in the encapsulation efficiency of resveratrol was observed with an increase in WPI and inulin concentration level [92]. In addition to the successful encapsulation of resveratrol, WPI/inulin NDSs had promising prebiotic effects on the probiotic strain *Lactobacillus acidophilus* ATCC 43121 [92]. In minimal media, the addition of WPI/inulin NDSs to the media led to an increase in the viability of probiotics and presented similar viability to free inulin (positive control). This implies that WPI/inulin NDS can not only be used for delivery of BCs but also exert potential prebiotic effects after consumption.

### 4.3. Off-Flavor Reduction

Omega-3 fatty acids, such as DHA and eicosapentaenoic acid (EPA), have various health-promoting activities, such as reduction of age-related and cardiovascular diseases [93,94]. However, the fortification of omega-3 fatty acids in foods is often difficult since they are poorly soluble in water and readily oxidized to form undesirable fish-like off-flavor compounds. It has been reported that the encapsulation of omega-3 fatty acids in whey protein-based NDSs, such as nanoparticles [22] and multiple nanoemulsions [55], resulted in a decrease in the off-flavor development compared with unencapsulated ones. When DHA was fortified in skim milk at a concentration of 0.25 g/L, the development of primary and secondary oxidation products of DHA and formation of off-flavor compounds, such as fishy like off-flavor, were increased during storage in skim milk [22]. However, as DHA was encapsulated in whey protein-based NDSs, the production of markers of DHA autoxidation and off-flavor formation were extensively decreased. Zimet and Livney [21] reported that the use of a *β*-lg/pectin complex was useful in enhancing the oxidative stability of DHA compared to unencapsulated DHA. This may be due to the immobilization and shielding effects of the protein/pectin complex, which can reduce the reactivity of DHA and access of oxidizing agent to DHA [21].

## 5. Conclusions

Owing to the various functional properties of whey proteins, such as their binding ability to hydrophobic BCs, gelation, and emulsifying properties, whey proteins can be used as an ideal delivery material for NDSs. Whey protein NDSs could enhance the physical and chemical stability and bioavailability of BCs. In particular, the physicochemical properties of whey protein-based NDSs, including size and surface charge, were easily modulated by the use of several manufacturing processes, such as conventional thermal gelation, mild heat-induced gelation, and chemical modification, which extensively affected the bioavailability of BCs, prebiotic effects, and off-flavor reduction of omega-3 fatty acids. Since food is complicated and matrices and food components interact with NDSs, which may alter physicochemical and functional properties of NDSs containing BCs, future studies should focus on their interactions with food components during the application of whey protein-based NDSs to foods.
